# Long-term cognitive outcomes after mild COVID-19, critical COVID-19, and non-COVID critical illness: a prospective cohort comparison

**DOI:** 10.1038/s41598-026-54890-6

**Published:** 2026-05-27

**Authors:** Vanessa Raeder, Anneke Quitschau, Anna Gorsler, Nadine Külzow, Maria Schroeder, Anne Pohrt, Irina Eckert, Christiana Franke

**Affiliations:** 1 Charité – Universitätsmedizin Berlin, corporate member of Freie Universität Berlin and Humboldt Universität zu Berlin, Department of Neurology and Experimental Neurology, Hindenburgdamm 30, 12203 Berlin, Germany; 2 Fachklinik Für Neurologische Frührehabilitation, Kliniken Beelitz GmbH, Beelitz-Heilstätten, Brandenburg, Germany; 3 Faculty of Health Sciences Brandenburg, Brandenburg Medical School Theodor Fontane, Brandenburg, Germany; 4 Charité – Universitätsmedizin Berlin, Corporate Member of Freie Universität Berlin and Humboldt-Universität Zu Berlin, Institute of Biometry and Clinical Epidemiology, Charitéplatz 1, 10117 Berlin, Germany

**Keywords:** Post COVID-19 condition, Cognitive impairment, Post-intensive care syndrome, Post-acute infection syndromes, Intensive care unit, Critical illness, Diseases, Health care, Medical research, Psychology, Psychology

## Abstract

Long-term cognitive impairment is a recognized sequela of the post-COVID-19 condition (PCC). While it is unclear how acute COVID-19 severity contributes to these lasting deficits, some evidence suggests that critical illness may lead to cognitive deficits similar to post-intensive care syndrome. This study examined long-term cognitive, mental, and physical health in patients with varying acute COVID-19 severity (mild to critical), alongside patients critically ill from non-COVID-19 causes. We conducted a bicentric prospective observational study comparing patients with PCC after mild COVID-19 (*n* = 30), those requiring ICU care for acute COVID-19 (*n* = 14), and patients with prolonged non-COVID-19 ICU stays (*n* = 7), all assessed ≥ 12 weeks post-onset. Comprehensive neuropsychological assessments were conducted alongside evaluations of physical impairments, psychiatric symptoms, fatigue and health-related quality of life. Overall cognition was comparable between the mild COVID-19 and ICU groups. However, the mild COVID-19 group experienced higher cognitive fatigue and lower memory satisfaction than both ICU groups, along with higher rates of anxiety (59% vs. 15%) and depression (38% vs. 15%), and reduced mental health-related quality of life compared to COV-ICU patients. Long-term cognitive impairment occurred in PCC patients and ICU survivors, irrespective of acute disease severity. Patients with mild COVID-19 reported greater long-term psychological distress.

*Trial registration*: This study was retrospectively registered at the German Clinical Trials Register, DRKS00025523 on 21 June 2021 (https://drks.de/search/de/trial/DRKS00025523).

**Supplementary Information:**

The online version contains supplementary material available at 10.1038/s41598-026-54890-6.

## Introduction

Coronavirus disease 2019 (COVID-19), caused by severe acute respiratory syndrome coronavirus 2 (SARS-CoV-2), has spread rapidly and continues to impact global public health^1,2^. As a multi-organ disease, the acute phase of COVID-19 can present with a wide spectrum of symptoms, ranging from mild, cold-like manifestations to more severe respiratory complications such as pneumonia and acute respiratory distress syndrome^3,4^. In severe cases, these complications may necessitate hospitalization in an intensive care unit (ICU) for critical management. COVID-19 may also present with extrapulmonary manifestations, including neurological symptoms, and can persist beyond the immediate acute clinical symptoms^3–5^.

Following acute COVID-19, some individuals may develop post-COVID-19 condition (PCC), defined as persistent or emerging symptoms at least 12 weeks after acute infection that cannot be otherwise explained^6^. PCC often manifests as persistent neurocognitive and neuropsychiatric symptoms, such as cognitive deficits, fatigue, and depression, which negatively affect health-related quality of life (HRQoL) and long-term recovery^6–8^. Notably, these symptoms can occur independently of the initial acute illness severity, affecting both individuals with mild cases managed at home and those who required intensive care^9,10^. Patients who experienced a mild acute phase frequently report persistent cognitive deficits and fatigue, underscoring the long-term impact of SARS-CoV-2 on brain health, and may lead patients with PCC to seek specialized care^10,11^. Patients with PCC experience various cognitive symptoms, including difficulty concentrating, finding the right words, memory gaps, attention deficits, and a sensation often described as ‘brain fog’^12–15^. Comprehensive neuropsychological assessments (NPA) can identify challenges in processing speed, executive function, verbal fluency, and memory^16^.

In addition to PCC, individuals recovering from critical COVID-19 may experience long-term symptoms stemming not only from the direct effects of SARS-CoV-2 but also from the consequences of critical illness and intensive care itself, known as post-intensive care syndrome (PICS)^9,17,18^. PICS is characterized by long-term functional deficits that persist beyond acute care hospitalization and is defined as a new or worsening change in physical, cognitive, or mental health that may arise during or after an ICU stay and that affects the HRQoL of survivors^9,19^. Risk factors for PICS include ICU-related factors (e.g., acute respiratory distress syndrome, delirium, sedation, sepsis) and pre-existing conditions^9^.

The convergence of these findings prompts a fundamental question: Do enduring neurological deficits after severe COVID-19 stem primarily from the virus, or from the broader toll of critical illness and intensive care? To answer this, we conducted a prospective, three-arm comparison study. We assessed objective and subjective cognitive impairment alongside other clinical characteristics at least 12 weeks after acute COVID-19 of varying severity. We compared PCC patients after mild COVID-19, COVID-19 patients who required intensive care, and patients with prolonged ICU stays due to critical illness polyneuropathy or myopathy, with primary pulmonary disease or other non-cerebral causes, as the control group^20^.

As described by Gorsler et al.^20^, patients with severe COVID-19 had longer ICU stays and a slightly higher complication burden; severe acute respiratory distress syndrome was more frequent—leading to greater use of extracorporeal membrane oxygenation—and both thrombosis and infections with multidrug-resistant pathogens were more prevalent than in non-COVID-19 ICU patients, whereas delirium and sepsis were the most common complications in both groups^20^.

This article reports data from the fourth follow-up assessment point (FU4) and refers to previously published longitudinal data from baseline to the third follow-up in ICU patients, which suggested a substantial overlap in cognitive, mental, and physical impairments between severe COVID-19 survivors and non-COVID ICU patients^20^.

## Methods

### Study design

This bicentric prospective observational study was conducted at the neurological PCC outpatient clinic of Charité—Universitätsmedizin Berlin and the Kliniken Beelitz GmbH Brandenburg for neurorehabilitation. Ethical approval was granted by the State medical association Brandenburg (postCov-Cog-2021-2073-NIS ff) and the ethics committee of Charité—Universitätsmedizin Berlin (EA2/102/22). The study was conducted in accordance with the guidelines of the aforementioned institutions, the Declaration of Helsinki and follows the Strengthening the reporting of observational studies in epidemiology guidelines^21^. All participants, or their legal guardians, provided their written informed consent.

At the neurorehabilitation clinic, critically ill patients with COVID-19 (COV-ICU) and without COVID-19 (non-COV-ICU) after ICU treatment underwent NPA, among other tests, in an inpatient setting^20^. The FU4, conducted at least 12 weeks after discharge from the rehabilitation clinic, is the centerpiece of this analysis and took place in the neurological PCC outpatient clinic between September 2021 and November 2023. Additionally, patients presenting to our PCC outpatient clinic with self-reported PCC symptoms following a mild course of acute COVID-19 (mild-PCC) were included in FU4. Eligibility criteria included age ≥ 18, SARS-CoV-2 infection (assessed by polymerase chain reaction testing) ≥ 12 weeks prior, domestic quarantine, and absence of exclusion criteria (e.g., condition after cardiopulmonary resuscitation, hepatic encephalopathy, and pre-existing structural brain damage, severe psychiatric or neurological disorders)^20^. The study size in this analysis was determined by the number of participants available for follow-up and eligible during the study period, respectively.

Cognitive function, the primary endpoint of our study, included verbal and visual memory, attentional performance such as information processing speed, tonic and phasic alertness, divided attention, and executive functions (working memory, verbal fluency, inhibition, and cognitive flexibility). Secondary outcomes were self-reported memory function, psychiatric symptoms, fatigue, HRQoL, and physical impairment. Potential confounders like age, gender, education, and pre-existing conditions were considered in the study design and interpretation of results. Mental health, fatigue, HRQoL, subjective cognitive impairment, and medical history were examined as predictors of cognitive performance. To reduce bias, participants were enrolled prospectively, and assessments were identical across all groups using standardized, validated tests of cognitive function and well-established self-report instruments. Loss to follow-up was addressed through multiple contact attempts, the offer of home visits, and reimbursement for participation. Analyses were restricted to participants with available follow-up data.

### Neuropsychological assessment and self-reporting instruments

While the methodology largely follows Gorsler et al.^20^, the following measurements were reassessed in ICU groups and obtained from patients in the mild-PCC group: Montreal Cognitive Assessment (MoCA), Verbal Learning and Memory Test, Consortium to Establish a Registry for Alzheimer’s Disease (CERAD)—Word List, Wechsler Memory Scale revised—digit span, Trail Making Test A and B (TMT-A, TMT-B), phonological verbal fluency in the Regensburger Word Fluency Test (RWT), State-Trait-Anxiety-Depression-Inventory—state (STADI-S) and the European Quality of Life 5 Dimensions 5 Level (EQ-5D-5L)^20^.

Additional objective instruments were included to extend the scope of the assessment (see Supplementary file 1).

Visual memory was assessed with either the CERAD—Figures delayed recall in participants aged ≥ 50 years^22^ or the Wechsler Memory Scale IV—Visual memory II in younger patients^23^.

Attention was further evaluated with the computerized Test of Attentional Performance (TAP)^24^, specifically the subtests “Divided Attention” and “Alertness”. The “Alertness” subtest captures both tonic and phasic alertness, encompassing basal responsiveness and phasic arousal to a cue stimulus.

Executive functions were additionally assessed through semantic verbal fluency (RWT^25^), cognitive flexibility (TAP “Flexibility: Verbal”), and inhibitory control (TAP “Go/NoGo” and the Stroop—Color-Word-Interference-Test (Stroop-Test)^26^).

Furthermore, logical reasoning was tested with the Performance Testing System subtest 3^27^, and vocabulary was measured with either the Vocabulary Test^28^ or the Multiple-Choice Vocabulary Test—B^29^, as an indicator of premorbid intelligence^30^.

In addition, subjective self-reporting instruments like the Multifactorial Memory Questionnaire (MMQ)^31^ satisfaction, ability, and strategies subtests were applied. Mental health assessment was complemented by the revised Beck Depression Inventory—I (BDI-I)^32^ and the Hospital Anxiety and Depression Scale (HADS)^33^. Moreover, HRQoL was examined in greater detail using the Short Form 36^34^, which calculates a score for the physical and mental aspects of HRQoL. Further symptoms, also affecting the HRQoL, were recorded using the following instruments: the Fatigue Severity Scale (FSS)^35^ and the Fatigue Scale for Motor and Cognitive Functions (FSMC)^36^ for fatigue, the Pittsburgh Sleep Quality Index^37^ for sleep quality, and the Epworth Sleepiness Scale^38^ for daytime sleepiness^31^.

In FU4, current sociodemographic and medical data were collected through a semi-structured interview. Physical and functional status were reassessed using the modified Rankin Scale, the Barthel Index, the Early Rehabilitation Barthel Index, the extended Barthel Index, the Nursing Delirium Screening Scale and by clinical examination^20^. Mild-PCC patients underwent additional diagnostic procedures as part of a comprehensive baseline assessment, including cerebral magnetic resonance imaging (cMRI) and analysis of cerebrospinal fluid (CSF).

### Data analysis

All cognitive and self-report measures were analyzed according to the age-, gender-, and education-adjusted norms of the respective tests, where available. Raw scores not assigned to a specific norm value were estimated by linear interpolation. For comparability, the normative scores (T-scores, C-scores, Scaled Scores, Intelligence Quotient) were converted to z-scores by linear transformation, while percentile ranks were transformed using the area transformation method, based on the area proportion of a percentile rank in a standard normal distribution^39^. Scores were considered average if they were within one standard deviation below or above the mean of the normative sample (z-score ≥ –1 and ≤ 1). MoCA scores were considered impaired if < 26 points^40^. Self-reporting questionnaires and neurological scales were scored according to their manuals. The STADI-S was transformed to z-scores. We used the cut-off ≥ 8 points for determining noticeable elevation of anxiety and depression levels in the HADS^33^. To assess performance in the various cognitive domains, we calculated composite scores for memory, attention, executive functions, and an overall composite score^41^, by averaging the z-scores according to the assignments above (for more details, see Supplementary file 1) and as shown by Gorsler et al.^20^.

Standard descriptive statistics were used to compare socio-demographic, clinical, cognitive, and self-reported outcomes between groups. Data distribution was visually assessed via histograms and Q-Q plots. Considering the assessment of normality and small group sizes, non-parametric statistical tests were applied as appropriate (Kruskal–Wallis test, Mann–Whitney U test, and Fisher’s exact test). Pairwise post-hoc comparisons following three-group analyses were Bonferroni-corrected. Due to the exploratory nature of our study, all other analyses were not adjusted for multiple testing. Effect sizes were estimated using Phi/Cramer’s V or effect size r, as appropriate. Further statistical analysis was performed using Spearman’s rank correlation coefficient to calculate bivariate correlations. The two-sided significance level was set at *p* ≤ 0.05. All data analyses were performed using SPSS Statistics (IBM version 28.0).

## Results

**Fig. 1:**
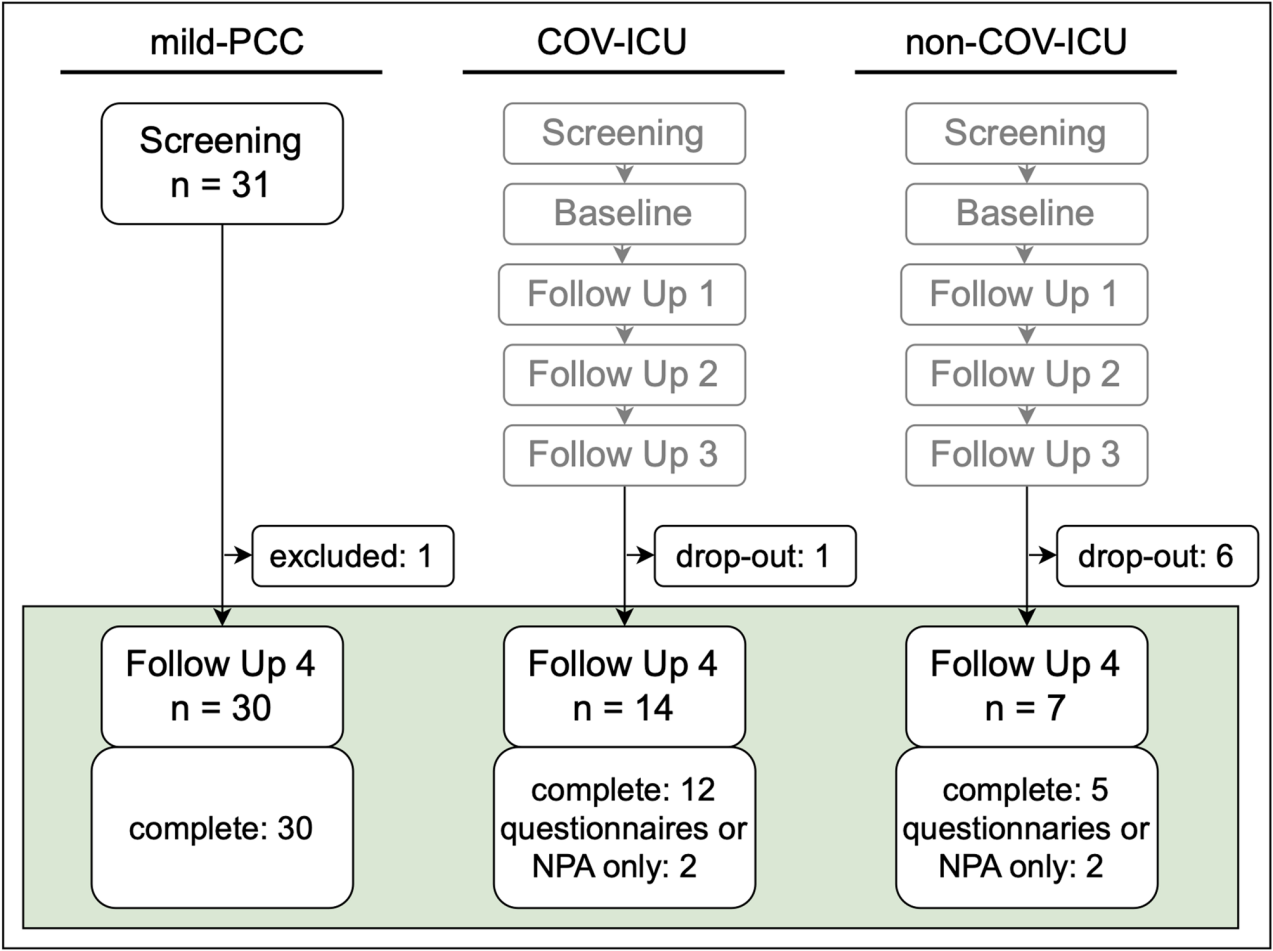
Overview of the timeline. The fourth follow-up is presented in the colored box. The grey-marked examinations Screening until the third follow-up took place in an inpatient setting in Kliniken Beelitz GmbH Brandenburg (Germany, neurorehabilitation clinic). For more details see Gorsler et al.^20^. *Abbreviations*: mild-PCC: post-COVID-19 condition group after mild initial course; COV-ICU: COVID-19 patients admitted to intensive care unit; non-COV-ICU: patients admitted to intensive care unit for critical conditions other than COVID-19; ICU: intensive care unit; NPA: neuropsychological assessment.

In the reported FU4, we studied a total of 51 patients (Fig. 1). FU4 was conducted in 14 COV-ICU patients and 7 non-COV-ICU patients. Home visits were conducted for two patients. Loss to follow-up could not be fully prevented due to death (n = 1), poor physical health, and personal reasons. Additionally, we enrolled 31 mild-PCC patients through the neurological post-COVID outpatient clinic. One person was excluded from this group retrospectively for not meeting inclusion criteria. The median interval between acute infection and FU4 was more than one year for the mild-PCC group and six months for the COV-ICU group (Table 1).

**Table 1:** Demographic and clinical characteristics.

Characteristic	mild-PCC (A)	COV-ICU (B)	non-COV-ICU (C)	*p*-value	Significant post-hoc analysis: *p*-value (groups)*	Effect size**
	N = 30	N = 14	N = 7			
Gender male/ female	6 (20.0%)/	10 (71.4%)/	4 (57.1%)/	0.002^1^	0.006 (AB)	Cramer’s V = 0.50
	24 (80.0%)	4 (28.6%)	3 (42.9%)			
Age in years	49 (43; 54)	63 (50; 67)	65 (57; 74)	< 0.001^2^	0.008 (AB)	r = 0.45
					0.002 (AC)	r = 0.56
Minimum–maximum	27–64	33–70	53–78			
Total education in years	16 (14; 18)	13 (12; 16)	13 (12; 19)	0.029^2^	0.031 (AB)	r = 0.39
Minimum–maximum	12–21	12–19	12–20			
Employment status:				< 0.001^1^	< 0.001 (AB)	Cramer’s V = 0.70
Working or job-seeking	21 (70.0%)	3 (21.4%)	1 (14.3%)			
- Reduced hours since acute illness	6 (20.0%)	1 (7.1%)	–			
Retired	-	8 (57.1%)	4 (57.1%)			
Inability to work	9 (30.0%)	3 (21.4%)	2 (28.6%)			
- New inability to work since acute illness	8 (26.7%)	3 (21.4%)	2 (28.6%)			
Living situation:				0.213^1^		
Alone	5 (16.7%)	2 (14.3%)	3 (42.9%)			
With partner/ family	25 (83.3%)	11 (78.6%)	4 (57.1%)			
Nursing home/ assisted living	–	1 (7.1%)	–			
Days between PCR and FU4	378 (285; 422)	184 (144; 293)	N/A	< 0.001^3^		r = 0.49
Minimum–maximum	170–596	126–564				
Days between ICU and FU4	N/A	180 (132; 290)	258 (158; 303)	0.360^3^		
Minimum–maximum		124–556	136–500			
Days between FU3 and FU4	N/A	83 (74; 113)	84 (57; 224)	0.856^3^		
Minimum–maximum		36–320	55–395			
Days at ICU	N/A	35 (25; 56)	24 (17; 44)	0.172^3^		
Minimum–maximum		15–119	15–62			
Days of ventilation	N/A	46 (23; 77)	52 (41; 56)	0.913^3^		
Minimum–maximum		8–209	12–77			

Data are presented as absolute values (percentage) or median (25th; 75th percentiles) and minimum–maximum. Significant *p*-values of three-group analyses are highlighted in bold. ^1^two-sided Fisher’s exact test. ^2^asymptotic, two-sided Kruskal–Wallis test. ^3^exact, two-sided Mann–Whitney-U test. *conducted with the according Bonferroni correction for multiple testing. In parentheses, the specific group comparison is shown. (AB) = mild-PCC vs. COV-ICU, (AC) = mild-PCC vs. non-COV-ICU, (BC) = COV-ICU vs. non-COV-ICU. **calculated were effect size r for Kruskal–Wallis test and Mann–Whitney-U test or Phi/ Cramer’s V for Fisher’s exact test. Suggestion for interpretation of effect sizes: 0.1 = weak, 0.3 = moderate, 0.5 = strong.

mild-PCC: post-COVID-19 condition group after mild initial course; COV-ICU: COVID-19 patients admitted to intensive care unit; non-COV-ICU: patients admitted to intensive care unit for critical conditions other than COVID-19; PCR: polymerase chain reaction; FU4: Fourth follow-up; FU3: Third follow-up; ICU: intensive care unit; N/A: not available/ not applicable.

### Characteristics

As indicated in Table 1, the mild-PCC group differed notably from the COV-ICU group. The mild-PCC group was younger (*p* = 0.008), more often female (*p* = 0.006), and had more years of education (*p* = 0.031) than the older, predominantly male COV-ICU group (Table 1). Employment rates were higher and retirement less common among mild-PCC patients (*p* < 0.001). The non-COV-ICU group did not differ from the COV-ICU group, but the median age was higher than in mild-PCC patients (*p* = 0.002) (Table 1).

### Neuropsychological assessment

The NPA (Table 2) showed no impairment in testing of logical reasoning or vocabulary on average, indicating normal levels of premorbid intelligence in all groups. Mean MoCA scores did not differ substantially between groups (*p* = 0.589), but approximately one third to half of each group demonstrated impairment in global cognition (Table 2). There were no significant differences in the combined NPA composite score (*p* = 0.689), which includes memory function, attention and executive functions, nor in the subcategory composite scores (Table 2).

**Table 2:** Neuropsychological assessment in the fourth follow-up.

Neuropsychological testing	mild-PCC		COV-ICU		non-COV-ICU		*p*-value
	n	z-score	n	z-score	n	z-score	
Vocabulary	29	1.13 ± 0.65	12	0.67 ± 0.89	6	1.57 ± 1.01	0.163^1^
Logical reasoning	30	0.61 ± 0.74	12	0.06 ± 0.81	5	0.46 ± 0.75	0.137^1^
	n	Points	n	Points	n	Points	
MoCA	30	26.1 ± 2.3	13	25.5 ± 2.6	6	25.0 ± 2.1	0.589^1^
n (%) with MoCA < 26 pt	30	11 (36.7)	13	6 (46.2)	6	3 (50.0)	0.761^2^
NPA subcategories							
	n	z-score	n	z-score	n	z-score	
NPA composite score	30	− 0.29 ± 0.55	13	− 0.29 ± 0.46	6	− 0.45 ± 0.61	0.689^1^
Memory – composite score	30	− 0.19 ± 0.82	13	− 0.21 ± 0.76	6	− 0.72 ± 0.83	0.317^1^
Verbal memory: Learning	30	0.05 ± 1.23	13	0.04 ± 1.01	6	− 1.24 ± 2.02	0.255^1^
Verbal memory: Consultation	30	− 0.36 ± 1.47	13	− 0.04 ± 1.04	6	− 0.53 ± 1.17	0.742^1^
Verbal memory: Recognition	30	− 0.45 ± 1.13	13	− 0.52 ± 1.22	5	− 1.16 ± 0.80	0.234^1^
Visual memory	30	− 0.01 ± 1.34	13	− 0.25 ± 1.29	6	− 1.27 ± 1.02	0.044^1^*
Short-term memory	30	− 0.17 ± 1.09	12	− 0.3 ± 1.05	5	0.67 ± 1.44	0.373^1^
n (%) with impairment in ≥ 1 memory test	30	19 (63.3)	13	6 (46.2)	6	4 (66.7)	0.579^2^
Attention – composite score	30	− 0.49 ± 0.68	13	− 0.60 ± 0.57	6	− 0.69 ± 0.66	0.599^1^
Information processing speed	30	− 0.46 ± 1.09	13	− 0.33 ± 0.91	6	− 0.55 ± 1.22	0.985^1^
Tonic alertness	30	− 0.63 ± 1.04	12	− 1.00 ± 0.62	5	− 1.26 ± 0.77	0.315^1^
Phasic alertness	30	− 0.46 ± 0.94	12	− 0.74 ± 0.75	5	− 0.71 ± 0.61	0.566^1^
Divided attention	30	− 0.41 ± 1.05	13	− 0.07 ± 1.03	6	− 0.78 ± 0.39	0.286^1^
n (%) with impairment in ≥ 1 attention test	30	19 (63.3)	13	9 (69.2)	6	4 (66.7)	1.000^2^
Executive functions – composite score	30	− 0.18 ± 0.58	13	− 0.06 ± 0.73	6	0.05 ± 0.72	0.867^1^
Working memory	30	− 0.29 ± 0.89	13	− 0.54 ± 1.10	6	0.29 ± 1.28	0.510^1^
Phonologic word fluency	30	0.01 ± 0.76	13	0.57 ± 1.11	6	0.62 ± 0.90	0.297^1^
Semantic word fluency	30	0.23 ± 0.79	13	0.72 ± 1.08	6	0.58 ± 0.74	0.382^1^
Flexibility (TMT-B)	30	− 0.49 ± 1.18	13	− 0.49 ± 1.21	6	− 0.72 ± 1.69	0.848^1^
Inhibition (Stroop-Test)	30	− 0.43 ± 0.58	11	− 0.27 ± 0.37	6	− 0.48 ± 0.97	0.689^1^
Flexibility (TAP)	4	0.51 ± 1.30	10	0.11 ± 1.52	4	− 0.18 ± 1.42	0.865^1^
Inhibition (TAP)	4	0.24 ± 0.57	9	− 0.08 ± 0.49	4	0.13 ± 0.48	0.544^1^
n (%) with impairment in ≥ 1 executive function test	30	15 (50.0)	13	8 (61.5)	6	4 (66.7)	0.705^2^

Group z-scores and points are given as mean ± standard deviation. Lower z-scores indicate worse cognitive performance, higher z-scores better performance. Significant *p*-values of three-group analyses are highlighted in bold. ^1^asymptotic, two-sided Kruskal–Wallis test. ^2^two-sided Fisher’s exact test. *post-hoc analysis showed a significant difference between the mild-PCC and non-COV-ICU group (adjusted *p*-value with Bonferroni correction = 0.041, effect size r = 0.41).

mild-PCC: post-COVID-19 condition group after mild initial course; COV-ICU: COVID-19 patients admitted to intensive care unit; non-COV-ICU: patients admitted to intensive care unit for critical condition other than COVID-19; MoCA: Montreal Cognitive Assessment; NPA: neuropsychological assessment; TMT-B: Trail Making Test B; Stroop-Test: Stroop Color-Word-Interference-Test; TAP: Test of Attentional Performance.

The assessment of memory revealed poorer visual memory performance in the non-COV-ICU group compared to mild-PCC patients (*p* = 0.044), while the remaining subtests did not differ between groups (Table 2). Additionally, group means of non-COV-ICU patients were below average range on verbal learning and verbal recognition, indicating a pronounced degree of impairment in these subtests.

In attention testing, tonic alertness was the most affected across all groups (Table 2). No significant differences were observed in subtests of attention between groups.

Executive function scores were within normal range for all groups (Table 2). Mild-PCC and non-COV-ICU patients were most impaired on cognitive flexibility (TMT-B) and inhibition (Stroop-Test). The COV-ICU group performed lowest on working memory and cognitive flexibility (TMT-B).

### Questionnaires

The mild-PCC group reported lower memory satisfaction and higher cognitive fatigue than the COV-ICU group (both *p* < 0.001) and non-COV-ICU group (*p* = 0.004 and *p* < 0.05, respectively). Furthermore, compared with the COV-ICU group, the mild-PCC group exhibited a higher prevalence of anxiety (STADI-S: *p* = 0.011, HADS: *p* = 0.004), depression symptoms (STADI-S: *p* = 0.030, HADS: *p* = 0.012, and BDI-I: *p* = 0.021), overall fatigue (FSS: *p* = 0.002 and FSMC: *p* = 0.001), and worse mental HRQoL (*p* < 0.001) (Table 3). Notably, the non-COV-ICU group reported a significantly lower general HRQoL compared to COV-ICU patients as assessed by the EQ-5D-5L visual analog scale (*p* = 0.038) (Table 3). The differences between groups were consistent throughout the various instruments assessing mental health and fatigue. The estimated effect sizes were moderate to strong for all significant results^42^.

**Table 3:** Self-reporting measures in the fourth follow-up.

Questionnaire	mild-PCC (A)		COV-ICU (B)		non-COV-ICU (C)		*p*-value	Significant post-hoc analysis: *p*-value (groups)*	Effect size**
	n	M ± SD	n	M ± SD	n	M ± SD			
Memory:									
MMQ – satisfaction (min.–max. points: 0–71)	29	29.5 ± 14.1	13	58.7 ± 11.5	6	56.3 ± 7.0	< 0.001	0.000 (AB)	0.74
								0.004 (AC)	0.55
MMQ – ability (min.–max. points: 0–80)	29	40.6 ± 14.1	13	59.5 ± 10.0	6	63.5 ± 5.8	< 0.001	0.001(AB)	0.57
								0.002 (AC)	0.58
MMQ – strategy (min.–max. points: 0–76)	29	38.3 ± 10.9	13	23.2 ± 15.0	6	19.7 ± 12.3	0.002	0.006 (AB)	0.43
								0.034 (AC)	0.39
Mental health:									
STADI-S Anxiety (z-score)	29	1.04 ± 0.98	13	0.08 ± 0.93	6	0.25 ± 1.19	0.008	0.011 (AB)	0.44
STADI-S Depression (z-score)	29	0.84 ± 1.01	13	− 0.15 ± 0.97	6	0.27 ± 1.01	0.028	0.030 (AB)	0.40
HADS – Depression (min.–max. points: 0–21)	29	6.0 ± 13.2	13	2.7 ± 2.8	5	6.6 ± 2.7	0.009	0.012 (AB)	0.44
HADS – Anxiety (min.–max. points: 0–21)	29	7.8 ± 3.4	13	3.6 ± 3.5	5	4.6 ± 3.1	0.003	0.004 (AB)	0.50
BDI-I (min–max. points: 0–63)	30	12.3 ± 6.7	13	6.4 ± 5.1	6	10.7 ± 5.0	0.023	0.021 (AB)	0.41
Sleep quality, daytime sleepiness:									
PSQI (min.–max. points: 0–21)	29	8.2 ± 3.3	13	6.3 ± 3.3	6	8.0 ± 5.0	0.166		
ESS (min.–max. points: 0–24)	30	10.0 ± 4.8	12	6.8 ± 3.9	6	8.3 ± 3.7	0.096		
Fatigue:									
FSS (min–max. points: 1–7)	30	5.5 ± 1.2	13	3.6 ± 1.7	6	4.8 ± 1.5	0.003	0.002 (AB)	0.52
FSMC – overall (min.–max. points: 20–100)	29	72.6 ± 18.0	13	45.2 ± 19.8	6	57.8 ± 17.5	0.001	0.001 (AB)	0.56
FSMC – cognitive (min.–max. points: 10–50)	29	37.5 ± 8.6	13	20.1 ± 10.5	6	25.0 ± 7.6	< 0.001	0.000 (AB)	0.64
								< 0.05 (AC)	0.40
FSMC – motor (min.–max. points: 10–50)	29	35.1 ± 10.0	13	25.1 ± 9.9	6	32.8 ± 10.6	0.024	0.019 (AB)	0.42
Quality of life:									
SF-36: PHS (z-score)	30	− 1.10 ± 1.20	13	− 1.10 ± 1.32	6	− 1.72 ± 0.45	0.334		
Physical functioning (z-score)	30	− 1.06 ± 1.49	13	− 1.38 ± 1.35	6	− 2.11 ± 0.71	0.078		
Physical role functioning (z-score)	30	− 1.72 ± 1.06	13	− 0.95 ± 1.03	6	− 1.59 ± 0.62	0.062		
Bodily pain (z-score)	30	− 0.19 ± 1.12	13	− 0.18 ± 1.07	6	− 0.23 ± 1.06	0.990		
General health perceptions (z-score)	30	− 1.07 ± 1.01	13	− 0.17 ± 0.95	6	− 1.24 ± 0.22	0.021	0.032 (AB)	0.39
SF-36: MHS (z-score)	29	− 1.33 ± 1.44	13	0.57 ± 0.60	6	− 0.65 ± 0.68	< 0.001	0.000 (AB)	0.66
Vitality (z-score)	29	− 1.63 ± 1.14	13	0.12 ± 1.01	6	− 1.28 ± 0.96	< 0.001	0.000 (AB)	0.63
Social role functioning (z-score)	29	− 1.58 ± 1.43	13	− 0.24 ± 1.02	6	− 1.49 ± 0.64	0.020	0.017 (AB)	0.43
Emotional role functioning (z-score)	29	− 1.17 ± 1.77	13	0.03 ± 0.76	6	− 0.96 ± 1.55	0.859		
Emotional wellbeing (z-score)	29	− 0.54 ± 1.20	13	0.72 ± 0.43	6	0.17 ± 0.68	0.001	0.001 (AB)	0.57
EQ-5D-5L Index (min.–max. points: -0.661–1)	29	0.81 ± 0.18	13	0.80 ± 0.2	6	0.57 ± 0.33	0.095		
EQ-5D-5L VAS (min.–max. points: 0–100)	28	62.0 ± 18.9	13	70.7 ± 21.7	6	47.5 ± 15.4	0.042	0.038 (BC)	0.57

All group data is given as mean ± standard deviation. Reported *p*-values are based on the asymptotic two-sided significance of the Kruskal–Wallis test. Significant *p*-values of three-group analyses are highlighted in bold. *conducted with the according Bonferroni correction for multiple testing. In parentheses the specific group comparison is shown. (AB) = mild-PCC vs. COV-ICU, (AC) = mild-PCC vs. non-COV-ICU, (BC) = COV-ICU vs. non-COV-ICU. **calculated was effect size r for Kruskal–Wallis test. Suggestion for interpretation of effect sizes: 0.1 = weak, 0.3 = moderate, 0.5 = strong.

mild-PCC: post-COVID-19 condition group after mild initial course; COV-ICU: COVID-19 patients admitted to intensive care unit; non-COV-ICU: patients admitted to intensive care unit for critical condition other than COVID-19; M ± SD: mean ± standard deviation; MMQ: Multifactorial Memory Questionnaire; min.–max.: minimum–maximum; STADI-S: State-Trait-Anxiety-Depression-Inventory: state; HADS: Hospital Anxiety and Depression Scale; BDI-I: Beck Depression Inventory: I; ESS: Epworth Sleepiness Scale; PSQI: Pittsburgh Sleep Quality Index; FSS: Fatigue Severity Scale; FSMC: Fatigue Scale for Motor and Cognitive Functions; SF-36: Short Form-36 Health Survey; PHS: physical health score; MHS: mental health score; EQ-5D-5L: European Quality of Life 5 Dimensions 5 Level; VAS: visual analog scale.

The STADI-S revealed elevated levels of anxiety and depression in 59% (n = 17) and 38% (n = 11) of mild-PCC patients, respectively, and in 15% (n = 2) of COV-ICU patients for both domains. Non-COV-ICU patients reported elevated levels of anxiety in 17% (n = 1) and depression in 33% (n = 2). In comparison, the HADS showed a similar degree of high anxiety (in 59% (n = 17) of mild-PCC patients, 15% (n = 2) of COV-ICU patients and 20% (n = 1) of non-COV-ICU patients), while elevated levels of depression were detected in only 31% (n = 9) of mild-PCC patients, in none of the COV-ICU patients, and in 20% (n = 1) of non-COV-ICU patients.

**Fig. 2:**
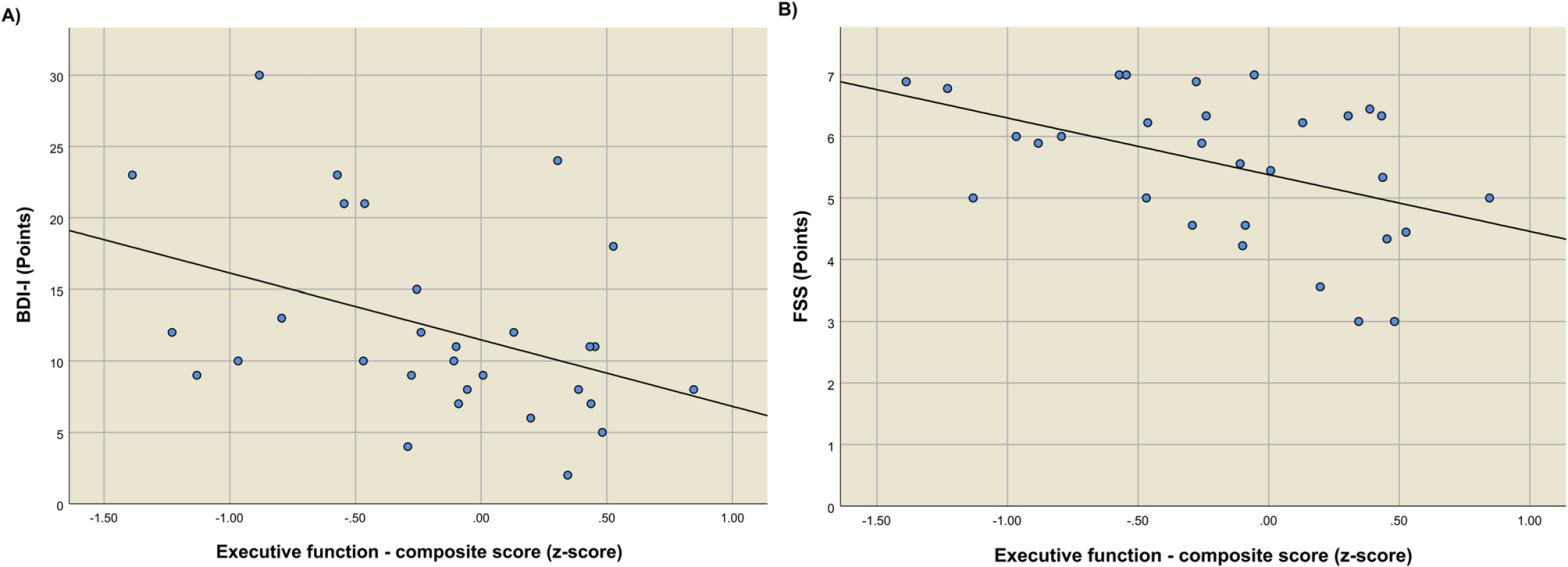
Scatter plots in the group of patients with post-COVID-19 condition after mild initial infection. (**a**) Scatter plot of the Beck Depression Inventory: I and the composite score for executive functions. Coefficient of determination R^2^ = 0.162. (**b**) Scatter plot of the Fatigue Severity Scale and the composite score for executive functions. Coefficient of determination R^2^ = 0.206. *Abbreviations*: BDI-I: Beck Depression Inventory: I; FSS: Fatigue Severity Scale.

In the mild-PCC group, a significant negative correlation was observed between performance on executive functions and depressive symptoms as measured by the BDI-I score, and the fatigue score in the FSS (Fig. 2), respectively (Spearman’s rank correlation coefficient (*p*-value): -0.42 (0.020) and -0.44 (0.015)). There were more symptoms of depression and fatigue in mild-PCC patients with poorer neuropsychological performance, which was not observed in the COV-ICU and non-COV-ICU group. Regarding the relation between subjective and objective cognitive performance, a significant positive correlation was found between memory satisfaction, as indicated by the MMQ, and the memory composite score in the mild-PCC group (Spearman’s rank correlation coefficient (*p*-value): 0.39 (0.039)), but not in the COV-ICU group and non-COV-ICU group. Notably, in the non-COV-ICU group, better cognitive performance, measured by the overall composite score, was significantly correlated with shorter duration of mechanical ventilation (Spearman’s rank correlation coefficient (*p*-value): -0.83 (0.042)), which was not significant in the COV-ICU group.

### Clinical assessment

The most common pre-existing condition was arterial hypertension across all groups (see Supplementary file 2 for detailed clinical data and Gorsler et al.^20^ for more information on ICU-groups). There were no significant differences in frequency of pre-existing conditions in post-hoc subgroup analysis; however, mild-PCC patients had a lower use of long-term medication than COV-ICU patients (p < 0.001) (Supplementary file 2). The frequency of complications during the initial ICU stay was comparable between both ICU groups (*p* = 0.667) (Supplementary file 2), but COV-ICU patients developed acute respiratory distress syndrome more frequently than non-COV-ICU patients (two-sided Fisher’s exact test: *p* = 0.018).

Regarding the neurological scores, the mild-PCC group was less impaired than both the COV-ICU and non-COV-ICU group, as reflected by the Early Rehabilitation Barthel Index (*p* = 0.044 and *p* = 0.001, respectively) (Supplementary file 2). The modified Rankin Scale showed scores ≥ 3 in 31% (n = 4) of the COV-ICU and 57% (n = 4) of non-COV-ICU patients, indicating middle to higher grade physical impairment, but in none of the mild-PCC patients.

Additionally, mild-PCC patients underwent more diagnostic procedures as part of their initial diagnostic work-up. 77% (n = 23) of patients got a cMRI, which showed no pathological signs in most patients, but an old cerebellar infarction and a symmetrical expansion of the parietal sulci in each one patient. CSF was analyzed in 53% (n = 16) of patients. There were no findings of autoantibodies in CSF, but autoantibodies against Myelin were detected in 19% (n = 3) of serum samples.

## Discussion

The present, prospective study examined cognitive, mental, and physical health outcomes in patients following mild and severe COVID-19 as well as in ICU patients with non-COVID pulmonary or other non-cerebral conditions at least 12 weeks prior. By including an ICU control group, the study provides a unique comparison of COVID-19-specific and general critical illness-related cognitive outcomes, which are crucial for the development of effective rehabilitation strategies. The overall cognitive performance did not differ across all groups, while the most pronounced deficits in mild-PCC and COV-ICU patients were observed in tests of attention. Notably, a considerable proportion of participants screened below the conventional MoCA cut-off (< 26), despite largely comparable composite scores in the detailed NPA (Table 2)^40^. Comparable rates have been reported in neurological post-COVID outpatient settings and in survivors of critical illness, indicating that screening positivity may reflect both the sensitivity of brief global screening and the symptom-enriched referral context rather than isolated cognitive decline^9,10,16,43^. Therefore, MoCA results in our cohort should be interpreted as screening signals and considered alongside domain-specific testing and concurrent symptoms such as fatigue and mood disturbances. Non-COV-ICU patients performed lowest in tests of memory; particularly scores of visual memory were lower than in mild-PCC patients. This direction is consistent with the PICS literature describing persistent memory and other cognitive deficits after critical illness^9,43^. Mild-PCC patients perceived their cognitive function to be more severely impaired and reported diminished mental health, along with a higher prevalence of fatigue, and lower mental HRQoL, compared to ICU patients. This pattern aligns with reports that subjective cognitive complaints in PCC are frequently accompanied by fatigue and affective symptoms and may exceed the degree of impairment captured by objective tests^7,44–46^. Further, our study provides follow-up data on the COV-ICU group and, while interpretability is limited, on the non-COV-ICU group at median 6 and 8.5 months post-ICU treatment, respectively, and offers insights into the recovery process that extends beyond the scope of early clinical rehabilitation. The COV-ICU and non-COV-ICU patients demonstrated persistent deficits, consistent with PICS literature^9,43^. Compared to pre-FU4 assessments^20^, both groups improved in information processing speed, indicating ongoing cognitive recovery, while attention deficits were found in two-thirds of both groups. These persistent deficits align with findings from the landmark BRAIN-ICU study by Pandharipande et al.^43^, demonstrating long-term cognitive impairment after critical illness up to 12 months post-discharge, comparable in magnitude to mild Alzheimer’s disease or traumatic brain injury. Our data suggest that attention and processing speed may remain vulnerable in these cohorts regardless of the specific precipitating cause. This vulnerability highlights the critical role of the ICU Liberation Bundle (ABCDEF) in mitigating PICS. While the bundle aims to reduce delirium and sedation, its ‘F’ component (Family engagement) was notably compromised during the pandemic by visitation restrictions, potentially compounding the long-term cognitive burden observed in our cohort^47^. Given that attention is the foundation for higher brain functions^48^, this may contribute to the deficits observed in executive functions throughout the assessments.

Regarding the long-term cognitive development in both ICU groups, executive functions were impaired in the majority of patients on the initial inpatient assessment, but in fewer patients at FU4^20^. This is consistent with PICS literature, where cognitive dysfunction after critical illness frequently involves executive abilities, among other domains^9^. Enhancements were noted in executive functions such as verbal fluency and working memory, while memory trajectories differed between groups. Overall, cognitive recovery may extend for several months, whereas attentional deficits may persist in our ICU cohorts irrespective of their COVID-19 status^9,20^.

Interestingly, COV-ICU patients did not perform worse than mild-PCC patients in the overall cognitive assessment despite the anticipated impact of PICS on cognitive performance^43^. This aligns with previous literature, as meta-analytic findings indicate no clear association between the initial disease severity and the development of cognitive impairment^7,44^. The higher frequency of fatigue and perceived cognitive impairment in mild-PCC patients may reflect selection bias from our neurological PCC outpatient clinic, to which patients present for cognitive symptoms and fatigue. Accordingly, mild-PCC patients reported lower perceptions of their memory function, mental health and mental HRQoL compared to COV-ICU patients. Patients with PCC frequently experience depressive symptoms and anxiety^44^, and depression is associated with an elevated risk of cognitive impairment^49^. A meta-analysis, including 784 currently depressed patients and 727 controls, demonstrated deficits in memory, executive functions and attention among patients with depression, which may also persist in remitted patients^49^. Fatigue symptoms, similar to those in chronic fatigue syndrome, may also negatively affect various areas of cognition^50^. In our study, lower executive function scores in mild-PCC patients were associated with higher rates of depressive symptoms and fatigue. Previous studies suggest associations between these symptoms, particularly fatigue, and cognitive dysfunction in PCC patients^45,46^. Furthermore, the ability to meet the demands of daily life activities, including cognitive requirements, may significantly impact mental health and HRQoL. In our study, mild-PCC patients were younger than participants of both ICU groups and less likely to be retired compared to COV-ICU patients. Consequently, mild-PCC patients were more frequently unable to resume work or had to reduce their working hours following SARS-CoV-2 infection, highlighting the substantial impact of PCC on individual lives and its broader societal relevance.

These sociodemographic factors likely contribute to the paradoxically worse subjective outcomes observed in the Mild-PCC group compared to ICU survivors. As suggested by Pihlaja et al.^51^, subjective cognitive symptoms in PCC may often be more strongly associated with psychological and demographic factors than with objective deficits^51^. Beyond the biological impact of the virus, this discrepancy may stem from differing baselines and expectations. Unlike ICU survivors, for whom survival and basic functional recovery are often the primary milestones, patients after mild acute illness generally measure their recovery against a high pre-morbid baseline of professional and social functioning. The inability to meet these specific demands likely exacerbates psychological distress and lowers HRQoL, even in the absence of severe objective cognitive deficits. Conversely, we observed a significant correlation between lower memory satisfaction and reduced memory performance in patients following mild COVID-19, indicating that subjective cognitive impairment may at least partially reflect on measurable deficits within this group. This was not observed among ICU survivors, which may be explained by the higher level of memory satisfaction in both ICU groups, potentially influenced by previously mentioned factors, and limited sample sizes.

The data quality was high, although several limitations should be considered. The specific analyses were limited in statistical power due to the small number of participants in the COV-ICU and non-COV-ICU groups. The prospective study design was conducted under dynamically changing conditions, regarding emerging SARS-CoV-2 variants, fluctuating infection rates, and increasing vaccinations, affecting recruitment over time in the ICU groups. The high number of non-COV-ICU patients lost to FU4 and the variability in assessment timing beyond 12 weeks post-infection may limit the interpretability of data, possibly introducing attrition and measurement bias, respectively. Furthermore, MoCA is a screening instrument and the commonly used cut-off (< 26) prioritizes sensitivity, which may overestimate impairment depending on demographic and setting-related factors^40^. Accordingly, MoCA-based impairment in our cohort should be interpreted cautiously and in conjunction with the comprehensive, norm-adjusted NPA results. Significant demographic differences emerged between subgroups, concerning potential confounders like age, gender and education. Mild-PCC patients were predominantly female, while particularly COV-ICU patients were mainly male. This is consistent with differing risk factors for PCC and severe COVID-19, as the risk for PCC is higher in females and increasing with age^52^, whereas the risk for severe acute COVID-19 is higher in males and with advancing age^53^. To mitigate confounding, all NPA subtests were adjusted for age, gender and education, where available; however, there may be residual confounding. While the FU4 data provide information about the recovery process of both the COV-ICU and non-COV-ICU groups, a longitudinal comparison for the mild-PCC group is not available, compromising conclusions regarding the trajectory of PCC following mild COVID-19.

Recruitment through two different institutions introduces a risk of bias. Considering this factor alongside the small and heterogeneous cohorts, potential selection, measurement, and attrition bias, as well as limited longitudinal data, the generalizability of our findings regarding cognitive performance following mild to severe COVID-19 and critical illness is restricted.

To distinguish shared from condition-specific outcomes, future research will benefit from comparing our findings—particularly for the mild COVID-19 group—with cohorts recovering from other infectious diseases (e.g., Borrelia infection), for which various neuropsychiatric manifestations and cognitive impairment have been reported^54^ and from extending analyses to additional post-acute infection syndromes. Further studies in larger cohorts are needed; however, the recruitment of control groups without a history of SARS-CoV-2 infection may be challenging. Furthermore, questions regarding the pathophysiology of post-acute infection syndromes, including PCC, remain. Different mechanisms may contribute, such as autoimmunity, direct organ damage through the initial infection, chronic reaction to persistent pathogens, and effects on the gut-brain axis^55^. Our diagnostic results align with recent evidence suggesting functional rather than structural pathology in PCC. Consistent with Franke et al.^58^, we detected serum myelin autoantibodies (19%) without intrathecal correlates, ruling out active autoimmune encephalitis^56^. Similarly, unremarkable cMRI findings in our cohort support findings suggesting that neurological symptoms might arise from metabolic or hemodynamic adaptations, including altered brain oxygenation, rather than macroscopic structural damage^57^.

In conclusion, our exploratory study indicates that patients recovering from mild or severe COVID-19 exhibit similar outcomes in cognitive functions at least 12 weeks post-infection. However, patients with PCC often experience impaired mental health and reduced HRQoL. This may have a greater impact on patients who have experienced a mild course of COVID-19 than on those who have required intensive care. The high prevalence of incapacity to work in this group, persisting even a year after the initial infection, underscores the significance of PCC and its healthcare burden. From a clinical perspective, our findings suggest that diagnostic workups for PCC should not rely solely on objective cognitive performance but should extend to screening for mental health symptoms and fatigue, as these factors appear to contribute significantly to the subjective burden and work incapacity observed in our cohort, particularly among the younger, working-age patients predominantly found in the mild-PCC group. Consequently, rehabilitation strategies may benefit from a multimodal approach combining cognitive training with a focus on attention, psychological interventions, and fatigue management to support vocational reintegration. The potential of SARS-CoV-2 to cause long-term post-acute sequelae is not unique. Previous research has shown that certain acute infections can be associated with persistent, poorly understood chronic symptoms in a subset of patients, which are referred to as post-acute infection syndromes^55^. These parallels highlight the critical importance of advancing fundamental research to unravel the mechanisms underlying these chronic conditions and highlight the urgency of identifying new therapies and preventive strategies. While current intervention studies investigating the efficacy of treatments such as methylprednisolone for cognitive impairment in PCC^58^ offer promising insights, current therapeutic options remain limited. This indicates the necessity for further research in this field.

## Supplementary Information

Below is the link to the electronic supplementary material.

Supplementary Material 1

Supplementary Material 2

## Data Availability

The datasets used and/or analyzed during the current study are available from the corresponding author on reasonable request.
