# Supplementary material for: Long-term cognitive outcomes after mild COVID-19, critical COVID-19, and non-COVID critical illness: a prospective cohort comparison
Source: Sci Rep. 2026 May 27;16:16453. doi: 10.1038/s41598-026-54890-6 (PMC13216524; doi:10.1038/s41598-026-54890-6)
Supplement: Supplementary file 1 — Supplementary Material 1 [file 41598_2026_54890_MOESM1_ESM.pdf]

Supplementary file 1: Neuropsychological assessment in Follow-Up 4.

	**Recurring***	**New****
**Memory**	VLMT	CERAD – Figures
	CERAD Word List	WMS-IV – Visual memory II
	WMS-r – digit span forward	
**Attention**	Trail Making Test A	TAP – Alertness
		TAP – Divided Attention
**Executive functions**	RWT – phonological verbal fluency	RWT – semantic verbal fluency
	Trail Making Test B	TAP – Flexibility: Verbal
	WMS-r – digit span backward	TAP – Go/NoGo
		Stroop-Test
**Other**	MoCA	LPS-3
		WST
		MWT-B

*Tests, which were previously used during the inpatient assessments and re-applied at Follow-Up 4. **Tests, which were newly added in Follow-Up 4.

Abbreviations: VLMT – Verbal Learning and Memory Test; CERAD – Consortium to Establish a Registry for Alzheimer's Disease; WMS-IV – Wechsler Memory Scale IV; WMS-r – Wechsler Memory Scale revised; TAP – Test of Attentional Performance; RWT – Regensburger Word Fluency Test; Stroop-Test – Stroop Color-Word-Interference-Test; MoCA – Montreal Cognitive Assessment; LPS-3 – Performance Testing System subtest 3, WST – Vocabulary Test/ "Wortschatztest"; MWT-B – Multiple-Choice Vocabulary Test–B.
