# Supplementary material for: Long-term cognitive outcomes after mild COVID-19, critical COVID-19, and non-COVID critical illness: a prospective cohort comparison
Source: Sci Rep. 2026 May 27;16:16453. doi: 10.1038/s41598-026-54890-6 (PMC13216524; doi:10.1038/s41598-026-54890-6)
Supplement: Supplementary file 2 — Supplementary Material 2 [file 41598_2026_54890_MOESM2_ESM.pdf]

Supplementary file 2: Clinical assessment at Follow-Up 4.

**Clinical assessment**	**mild-PCC (A) N=30**	**COV-ICU (B) N=14**	**non-COV-ICU (C) N=7**	**p-value**
	n (%)	n (%)	n (%)	
Medication: yes	10 (33.3%)	13 (92.9%)	6 (85.7%)	<0.001[1]
Post-hoc test for (AB) [Effect size]				<0.001 [0.56[a]]
Anticoagulation	1 (3.3%)	7 (50.0%)	4 (57.1%)	
Antidepressants	5 (16.7%)	0	2 (28.6%)	
Sedatives	0	0	0	
Antihypertensives	4 (13.3%)	9 (64.3%)	6 (85.7%)	
Neuroleptics	0	0	0	
Antiepileptics	3 (10.0%)	3 (21.4%)	1 (14.3%)	
Anticholinergics	0	0	0	
Medical devices: yes	0	1 (7.1%)	1 (14.3%)	0.165[1]
Nasogastric feeding tube	0	0	0	
PEG	0	0	0	
Tracheostomy	0	0	0	
Urinary catheter	0	1 (7.1%)	0	
Invasive ventilation >6 h/d	0	0	0	
Noninvasive ventilation	0	0	1 (14.3%)	
		**Comorbidities**		
Internal comorbidities: yes	11 (36.7%)	11 (78.6%)	6 (85.7%)	0.008[1]
Diabetes	0	5 (35.7%)	2 (28.6%)	
Hypertension	5 (16.7%)	9 (64.3%)	5 (71.4%)	
Renal insufficiency	0	1 (7.1%)	0	
COPD/Asthma	2 (6.7%)	0	2 (28.6%)	
Atrial fibrillation	0	3 (21.4%)	1 (14.3%)	
Heart failure	0	1 (7.1%)	3 (42.9%)	
Obesity	1 (3.3%)	5 (35.7%)	1 (14.3%)	
CHD	0	1 (7.1%)	2 (28.6%)	
Other internal medical condition	11 (36.7%)	6 (42.9%)	4 (57.1%)	
Anxiety, depression	6 (20.0%)	0	0	0.147[1]
Migraine	5 (16.7%)	0	0	0.243[1]
Complications on ICU: yes	N/A	13 (92.9%)	7 (100%)	0.667[1]
Thrombosis		5 (35.7%)	0	
Pulmonary embolism		3 (21.4%)	0	
Myocardial infarction		0	1 (14.3%)	
Dialysis		5 (35.7%)	1 (14.3%)	
Delirium		11 (78.6%)	5 (71.4%)	
ARDS		8 (57.1%)	0	
Epileptic seizure		3 (21.4%)	0	
Sepsis		8 (57.1%)	3 (42.9%)	
Liver failure		1 (7.1%)	0	
Anxiety, depression		1 (7.1%)	2 (28.6%)	
Multidrug-resistant pathogens		4 (28.6%)	0	
		**Neurological scores**		
	**Mild-PCC (A)**	**COV-ICU (B)**	**Non-COV-ICU (C)**	**p-value**

	N	M ± SD	N	M ± SD	N	M ± SD	
ERBI	30	100.0 ± 0.0	14	92.5 ± 18.7	7	86.4 ± 23.8	<0.001[2]
Post-hoc test for (AB) [Effect size]							0.044 [0.37[b]]
Post-hoc test for (AC) [Effect size]							0.001 [0.59[b]]
mRS	30	0.0 ± 0.2	13	1.2 ± 1.6	7	2.4 ± 1.3	<0.001[2]
Post-hoc test for (AC) [Effect size]							<0.001 [0.75[b]]
EBI	30	90.0 ± 0.0	13	90.0 ± 0.0	6	90.0 ± 0.0	
NuDESC	30	0.0 ± 0.0	13	0.0 ± 0.0	6	0.0 ± 0.0	
**Neurological examination**							
	N	n (%)	N	n (%)	N	n (%)	
Cranial nerves abnormal:	30	6 (20.0%)	13	4 (30.8%)	6	2 (33.3%)	
- Anosmia/ Hyposmia/ Parosmia	30	6 (20.0%)	13	3 (23.1%)	6	0	
- Hypogeusia/ Dysgeusia	30	5 (16.7%)	11	2 (18.2%)	6	1 (16.7%)	
Muscle tone abnormal	30	0	10	2 (20.0%)	4	0	
Reflexes abnormal	30	0	8	2 (25.0%)	3	1 (33.3%)	
Sensation abnormal	30	7 (23.3%)	12	10 (83.3%)	4	2 (50.0%)	
Extrapyramidal dysfunction	30	0	12	0	6	0	
Cerebellar examination abnormal	30	0	11	1 (9.1%)	4	1 (25.0%)	
Stance/ gait abnormal:	30	3 (10.0%)	11	7 (63.6%)	5	5 (100.0%)	
- ≥4 steps without walking aid	30	0	11	2 (18.2%)	5	3 (60.0%)	
- Walking aid	30	0	11	1 (9.1%)	5	0	
- Wheel chair	30	0	11	1 (9.1%)	5	0	
- Other impairment*	30	3 (10.0%)	11	3 (27.3%)	5	2 (40.0%)	
Attention/ concentration abnormal	30	24 (80.0%)	13	0	6	1 (16.7%)	
Memory abnormal	30	16 (53.3%)	13	1 (7.7%)	6	0	

Adopted by Gorsler et al. (2024). Data is given as n (%) or mean ± standard deviation. All post-hoc tests are conducted with the according Bonferroni correction for multiple testing and are shown, if ≤0.05. [1] two-sided Fisher's exact test. [2] asymptotic, two-sided Kruskal-Wallis test. [a] Cramer's V. [b] effect size r. Suggestion for interpretation of effect sizes: 0.1 = weak, 0.3 = moderate, 0.5 = strong. *Abnormalities in stance or gait without fitting the categories above, such as instability or impaired balance during toetip gait, heel walk, or tightrope walk.

Abbreviations: mild-PCC – post-COVID-19 condition group after mild initial course; COV-ICU – COVID-19 patients admitted to intensive care unit; non-COV-ICU – patients admitted to intensive care unit for critical condition other than COVID-19; (AB) – mild-PCC vs. COV-ICU; (AC) – mild-PCC vs. non-COV-ICU; PEG – Percutaneous Endoscopic Gastrostomy, COPD – chronic obstructive pulmonary disease; CHD – coronary heart disease; ICU – intensive care unit; N/A – not available/ not applicable; ARDS – acute respiratory distress syndrome; ERBI – Early Rehabilitation Barthel Index; mRS – modified Rankin Scale; EBI – extended Barthel Index; NuDESC - Nursing Delirium Screening Scale.
